# Association of Angiotensin-Converting Enzyme Inhibitors and Angiotensin II Blockers With Severity of COVID-19: A Multicenter, Prospective Study

**DOI:** 10.1177/1074248420976279

**Published:** 2020-11-24

**Authors:** Hakeam A. Hakeam, Muhannad Alsemari, Zainab Al Duhailib, Leen Ghonem, Saad A. Alharbi, Eid Almutairy, Nader M. Bin Sheraim, Meshal Alsalhi, Ali Alhijji, Sara AlQahtani, Mohammed Khalid, Mazin Barry

**Affiliations:** 1 Pharmaceutical Care, 37852 King Faisal Specialist Hospital & Research Centre, Riyadh, Saudi Arabia; 2 College of Medicine, Alfaisal University, Riyadh, Saudi Arabia; 3 Department of Surgery, 37852 King Faisal Specialist Hospital & Research Centre, Riyadh, Saudi Arabia; 4 Department of Health Research Methods, Evidence and Impact, McMaster University, Hamilton, Ontario, Canada; 5 Critical Care Medicine, King Faisal Specialist Hospital & Research Centre, Riyadh, Saudi Arabia; 6 Department of Pharmacy, Clinical Pharmacy Services, 534884 King Saud University Medical City, Riyadh, Saudi Arabia; 7 Pharmacy Services, Buriadh Central Hospital, Buriadh, Saudi Arabia; 8 Pulmonary Medicine, 37852 King Faisal Specialist Hospital & Research Centre, Riyadh, Saudi Arabia; 9 Pharmacy Service, 430300 King Abdullah Bin Abdulaziz University Hospital, Riyadh, Saudi Arabia; 10 Division of Infectious Diseases, Department of Internal Medicine, College of Medicine, 191082 King Saud University, Saudi Arabia

**Keywords:** COVID-19, angiotensin-converting enzyme inhibitor, angiotensin II receptor blockers, SARS-CoV-2

## Abstract

**Background:**

Speculations whether treatment with angiotensin-converting enzyme inhibitors (ACE-I) or angiotensin II receptor blockers (ARB) predisposes to severe coronavirus disease 2019 (COVID-19) or worsens its outcomes. This study assessed the association of ACE-I/ARB therapy with the development of severe COVID-19.

**Methods:**

This multi-center, prospective study enrolled patients hospitalized for COVID-19 and receiving one or more antihypertensive agents to manage either hypertension or cardiovascular disease. ACE-I/ARB therapy associations with severe COVID-19 on the day of hospitalization, intensive care unit (ICU) admission, mechanical ventilation and in-hospital death on follow-up were tested using a multivariate logistic regression model adjusted for age, obesity, and chronic illnesses. The composite outcome of mechanical ventilation and death was examined using the adjusted Cox multivariate regression model.

**Results:**

Of 338 enrolled patients, 245 (72.4%) were using ACE-I/ARB on the day of hospital admission, and 197 continued ACE-I/ARB therapy during hospitalization. Ninety-eight (29%) patients had a severe COVID-19, which was not significantly associated with the use of ACE-I/ARB (OR 1.17, 95% CI 0.66-2.09; *P* = .57). Prehospitalization ACE-I/ARB therapy was not associated with ICU admission, mechanical ventilation, or in-hospital death. Continuing ACE-I/ARB therapy during hospitalization was associated with decreased mortality (OR 0.22, 95% CI 0.073-0.67; *P* = .008). ACE-I/ARB use was not associated with developing the composite outcome of mechanical ventilation and in-hospital death (HR 0.95, 95% CI 0.51-1.78; *P* = .87) versus not using ACE-I/ARB.

**Conclusion:**

Patients with hypertension or cardiovascular diseases receiving ACE-I/ARB therapy are not at increased risk for severe COVID-19 on admission to the hospital. ICU admission, mechanical ventilation, and mortality are not associated with ACE-I/ARB therapy. Maintaining ACE-I/ARB therapy during hospitalization for COVID-19 lowers the likelihood of death.

**Clinical Trial Registration:**

ClinicalTrials.gov, NCT4357535.

## Introduction

Coronavirus disease 2019 (COVID-19), is a global pandemic caused by the novel RNA virus named the severe acute respiratory syndrome coronavirus 2 (SARS-CoV-2).^1^ The disease was first identified in Wuhan City, China in December 2019.^2^ The clinical spectrum of COVID-19 ranges from asymptomatic to serious illness characterized by respiratory failure, with a mortality rate reaching as high as 7.2%.^3^ Analysis of initial reports from China showed that hypertension, cardiovascular diseases, and diabetes are highly prevalent in SARS-CoV-2 infected patients and are associated with poor outcomes.^2,3^ These conditions are commonly treated with a renin-angiotensin-system (RAS) antagonist such as an angiotensin-converting enzyme inhibitor (ACE-I) or an angiotensin II receptor blocker (ARB).

For entry into human cells and subsequent replication, SARS-CoV-2 attaches to membrane-bound angiotensin-converting enzyme 2 (ACE2) via the virion’s surface protein spikes.^4^ ACE2, a homologue of ACE, which counterbalances the classical angiotensin II axis of the RAS, by metabolizing angiotensin II into the angiotensin 1-7 peptide. The central role of ACE2 in SARS-CoV-2 pathogenicity raised the concern that ACE-I/ARB therapy might be contributing to the increased severity of COVID-19, observed in hypertensive patients.^5^ It has been postulated that ACE-I/ARB therapy upregulates ACE2 expression; thereby making more ACE2 substrates available for SARS-CoV-2 to access to the human body.^5,6^ Earlier animal studies reported 5 and 3 fold increases in ACE2 expression upon the administration of lisinopril and losartan, respectively.^7^ In hypertensive patients, higher urinary ACE2 levels were observed with ARB treatment compared to other antihypertensive agents.^8^

Clinical evidence demonstrating the harm of ACE-I/ARB therapy during the COVID-19 pandemic is lacking. Therefore, several medical organizations jointly advised against discontinuing ACE-I/ARB therapy during the COVID-19 pandemic in patients with heart failure, hypertension, or ischemic heart disease.^9^ That advice was accompanied by a call for urgent clinical studies to assess the theoretical interaction between ACE-I/ARB and COVID-19. Large population-based studies found no association of COVID-19 diagnosis and mortality in patients using ACE-I/ARBs.^10,11^ However, these studies analyzed data on RAS blockers from registries and prescription filling reports, which might not reflect the actual use of these agents. In this regard, the self-discontinuation of ACE-I/ARB therapy in fear of severe COVID-19 might be underreported in registries.^12^ Moreover, these aforementioned studies did not describe the characteristics of ACE-I/ARB therapy, including dosing and complications such as hyperkalemia. Therefore, to address these gaps in information, we conducted a prospective clinical study to assess the association of ACE-I/ARB use with the severity of COVID-19 in patients with hypertension and cardiovascular diseases.

## Methods

### Study Design and Participants

This multicenter, prospective, cohort study, was conducted at 2 tertiary hospitals in Riyadh, King Faisal Specialist Hospital & Research Centre, and King Khalid University Hospital, as well as at 2 secondary-care hospitals, King Abdullah Bin Abdulaziz University Hospital, Riyadh and Buriadh Central Hospital, Qassim. In these 4 hospitals, COVID-19 patients were primarily admitted through the emergency department. The study protocol was approved by the institutional review board of each participating site. All consecutive patients aged ≥18 years, with a positive COVID-19 PCR assay obtained from nasopharyngeal or oropharyngeal swabs, and admitted to the hospital between May 10 and July 1, 2020 were screened for enrolment. We prospectively identified patients with hypertension or cardiovascular diseases and who were receiving one or more antihypertensive agents from the following groups: ACE-I, ARB, calcium channel blockers, β-blockers, and thiazides. The exclusion criteria included pregnancy, no current antihypertensive treatment on presentation to the hospital, receipt of chemotherapy within 4 weeks before hospital admission, and transfer from another hospital for COVID-19 treatment. Eligible patients were verbally consented for enrollment in the study upon hospital admission, using hospital telephones to minimize physical contact with the investigators.

### Procedure

On a daily basis, hospital admission logs were reviewed for eligible patients. Data were prospectively collected from the day of hospital admission and updated as soon as outcomes had been identified. Any missing or uncertain data were clarified through communication with the involved health-care providers or patients and their families. The obtained data included patients’ demographics, vital signs, smoking status, history of chronic illnesses, and symptoms of COVID-19 with their onsets. Complete blood counts and serum or plasma levels of the following biochemical studies were obtained within 24 hours of hospital admission: high-sensitivity C-reactive protein (CRP), D-Dimer, ferritin, creatinine kinase, creatinine, and potassium. Antihypertensive agent type and dose were obtained from each patient’s medical record and compliance was confirmed by the patient or a relative interview. During hospitalization, we prospectively monitored and recorded the initiation of the following management interventions: continuation of ACE-I/ARB therapy, type of oxygen therapy, vasopressor use, renal replacement therapy, and extracorporeal membrane oxygenation (ECMO) use (for patients admitted to King Faisal Specialist Hospital & Research Centre). On the day of admission to the intensve care unit (ICU), respiratory rate and mean arterial blood pressure (MAP), were recorded. The Sequential Organ Failure Assessment (SOFA) score and the ratio of partial pressure of oxygen in arterial blood to the fractional concentration of oxygen in inspired air (PaO_2_/FiO_2_) were determined. Patients developing acute respiratory distress syndrome (ARDS) and acute kidney injury (AKI) were identified and the severity was recorded. All patients received the standard of care treatment at the time of hospitalization according to the COVID-19 management guidelines of King Faisal Specialist Hospital & Research Centre and the Saudi Ministry of Health Guidelines for management of COVID-19 (e-Appendix, page 10). Patients were followed until discharged from the hospital or death. July 31, 2020, was the final day for follow-up.

### Study Outcomes

The primary study endpoint was the rate of developing severe COVID-19 on the day of hospitalization. The key secondary outcome was a composite of mechanical ventilation and in-hospital death. Other secondary outcomes were the rates of ICU admission, mechanical ventilation, and in-hospital death, as well as frequencies of developing hypo- or hyperkalemia, hyperinflammation, and AKI.

### Definitions

COVID-19 severity (either severe or non-severe) at the time of hospital admission was defined according to the World Health Organization (WHO) interim guidance.^13^ We adopted a composite outcome of mechanical ventilation and in-hospital death from previous studies to assess the severity of COVID-19.^14^ Serious vital signs were defined as: O_2_ saturation of <94%, respiratory rate of >30 breaths per min, MAP <65 mmHg or fever (temperature >38 ^o^C). Serum potassium was defined as hypokalemia or hyperkalemia when levels at <3.5 mmol/L or >5.2 mmol/L, respectively. Obesity was defined as a body mass index of ≥30 kg/m^2^. AKI was defined according to the Kidney Disease Improving Global Outcomes criteria.^15^ The following laboratory results were indicative of COVID-19 hyperinflammation state: ferritin level >800 ng/mL, CRP level >41.8 mg/L, D-Dimer level >1 µg/mL.^16^ ARDS was defined and classified according to the Berlin definition. In-hospital discontinuation of ACE-I/ARB was defined as >48 hours stop of therapy during a non-ICU stay. High dose ACE-I/ARB was defined as the maximum dose of each agent according to the US Food and Drug Administration approved doses.

### Statistical Analysis

Continuous variables are expressed using the mean and standard deviation or the median and interquartile range and were compared using the Welch *t*-test or the Mann-Whitney *U* test. Categorical variables are summarized as counts and percentages and examined using the χ^2^ test or Fisher’s test.

Associations of using ACE-I/ARB, or ACE-I alone, or ARB alone with the primary and secondary outcomes were tested using univariate and multivariate logistic regression to estimate the odds ratios (OR) and 95% confidence intervals (CI). We estimated the hazard ratios (HR) and 95% CI for the composite outcome of mechanical ventilation and death using Cox proportional-hazards models. We measured time to event in days from the date of hospital admission. For the multivariate logistic and Cox regressions, we created a model that was adjusted for the following independent variables (covariates) known to be associated with COVID-19 severity and mortality: age, obesity, and chronic illness, including hypertension, cardiovascular diseases, and diabetes.^2,3^

We tested for correlations between ACE-I and ARB doses and COVID-19 severity using the Spearman’s correlation test. Statistical significance was defined as a 2-sided *P* < .05. All statistics were performed using SPSS, version 20.0 IBM.

## Results

Of 1609 adult patients hospitalized with confirmed COVID-19 during the study period, 338 patients were enrolled. A total of 388 patients were considered for inclusion, but 7 denied consent to participate, and 43 additional patients were excluded for the following reasons: 14 stopped ACE-I/ARB therapy before hospitalization in fear of COVID-19 effect, 13 were referred from other hospitals, 8 were pregnant women, and 8 received chemotherapy within 4 weeks of COVID-19 diagnosis. On the day of hospitalization, 245 (72.5%) patients were using ACE-I/ARB, whilst 93 (27.5%) patients were using non-ACE-I/ARB antihypertensive agents, including calcium channel blockers, β-blockers, or thiazides. Classified according to the age decade, the largest number of patients was in the sixth decade. Users of ACE-I/ARB had a lower rate of chronic kidney disease (15.1%) compared with non-users (24.7%, *P* = .039) and a higher concomitant thiazide use (19.6% vs. 3.2%, *P* < .001). The other clinical characteristics and demographics were similar between ACE-I/ARB users and non-users, Table 1. By July 31, 2020, 331 (97.9%) patients had completed their hospital course (either discharged or died). This date allowed for 4 weeks of the follow-up period for the last patients enrolled on July 01, 2020. (e-Appendix, page 1, for details and the distribution of COVID-19 signs and symptoms at each hospital)

**Table 1.** Demographics and Clinical Characteristics of the Study Cohort Assessed According to the Use of ACE-I/ARB Therapy.

	Study cohort, n = 338	ACE-I/ARB, n = 245	Non-ACE-I/ARB, n = 93	*P* value
Sex				.86
Men	201 (59%)	145 (59.2%)	56 (60.2%)	
Women	137 (40.5%)	100 (40.8%)	37 (39.8%)	
Age, years	60.8 ± 13.5	59.7 ± 13.5	63.0 ± 13.4	.81
Age by decade				.66
<40 years	23 (6.8%)	18 (7.3%)	5 (5.4%)	
40-49 years	42 (12.4%)	33 (13.5%)	9 (9.7%)	
50-59 years	91 (26.9%)	68 (27.8%)	23 (24.7%)	
60-69 years	84 (24.9%)	60 (24.5%)	24 (25.8%)	
70-79 years	74 (21.9%)	51 (20.8%)	23 (24.7%)	
80-89 years	19 (5.6%)	11 (4.5%)	8 (8.6%)	
>90 years	5 (1.5%)	4 (1.6%)	1 (1.1%)	
Weight, kg	80.1 ± 16.4	80.6 ± 15.8	78.9 ± 17.8	.41
BMI, kg/m^2^	29.9 ± 6.3	30.0 ± 5.9	29.6 ±7.3	.11
Obese				.88
BMI < 30 kg/m^2^	202 (59.8%)	147 (60%)	55 (59.1%)	
BMI ≥30 kg/m^2^	136 (40.2%)	98 (40%)	38 (40.9%)	
Smoking, current or former	60 (17.8%)	48 (19.6%)	12 (12.9%)	.16
Chronic illnesses				
Median	2 (2-3)	2 (2-4)	2 (2-3)	.40
Hypertension	317 (93.8%)	230 (93.9%)	87 (93.5%)	.91
Diabetes	214 (63.3%)	156 (63.7%)	58 (62.4%)	.82
Heart failure	51 (15.1%)	40 (16.3%)	11 (11.8%)	.30
Ischemic heart disease	58 (17.2%)	43 (17.6%)	15 (16.1%)	.75
Stroke	20 (5.9%)	74 (30.2%)	26 (28%)	.44
Asthma	26 (7.7%)	16 (6.5%)	10 (10.8%)	.19
COPD	10 (3%)	7 (2.9%)	3 (3.2%)	.85
Dyslipidemia	119 (35.2%)	82 (33.5%)	37 (39.8%)	.27
Chronic kidney disease	60 (17.8%)	37 (15.1%)	23 (24.7%)	.03
Hemodialysis	8 (2.3%)	3 (1.22%)	5 (5.3%)	.11
Thiazide use	51 (15.0%)	48 (19.6%)	3 (3.2%)	<.0001
COVID-19 related symptoms				
Onset of illness before hospitalization , days	3 (2-7)	3 (2-7)	4 (2-7)	.25
Number of experienced symptoms	2 (1-2)	2 (1-2)	2 (1-2)	.24

ACE-I, angiotensin-converting enzyme inhibitor; ARB, angiotensin II receptor blockers; BMI, body mass index; COPD, chronic obstructive pulmonary disease.

Medians are presented as IQR.

### COVID-19 Severity and Outcomes on Admission Day

Amongst the study cohort, 98 (29%) patients met the WHO criteria for severe COVID-19 on the day of hospitalization. On univariate analysis, ACE-I/ARB use was not associated with developing severe COVID-19 (OR 1.07, 95% CI 0.63 -1.82). When examined separately, neither ACE-I nor ARB use was a risk factor for developing severe COVID-19 or serious vital signs of COVID-19 (Table 2). Assessed with the adjusted multivariate regression model, severe COVID-19 was not associated with using ACE-I/ARB (OR 1.17, 95% CI 0.66-2.09, *P* = .57); ACE-I (OR 1.36, 95% CI 0.77-2.42, *P* = .25); or ARB (OR 0.88, 95% CI 0.53-1.47, *P* = .63). Moreover, ACE-I/ARB therapy was not associated with increased risk for oxygen therapy or admission to the ICU within 24 hours of hospitalization. None of the patients in the entire cohort died within 24 hours of hospitalization.

**Table 2.** Univariate Regression Analysis and Odds of COVID-19 Severity Outcomes According to ACE-I and ARB Use on Admission to Hospital.

	ACE-I/ARB, N = 245, OR (95% CI)	ACE-I, N = 90, OR (95% CI)	ARB, N = 155, OR (95% CI)
COVID-19 severity*	1.07 (0.63-1.82)	1.14 (0.67-1.94)	0.93 (0.58-1.49)
Serious vital signs			
Respiratory rate >30 breaths/min	1.13 (0.57-2.23)	0.72 (0.35-1.48)	1.37 (0.75-2.5)
Fever	1.21 (0.74-1.95)	1.07 (0.66-1.73)	1.12 (0.73-1.73)
Mean arterial blood pressure <65 mmHg	1.87 (0.61-5.65)	0.97 (0.37-2.54)	1.56 (0.66-3.67)
O_2_ saturation <94%	0.84 (0.52-1.36)	0.79 (0.49-1.29)	0.92 (0.66-1.56)
Loss of conscious and dizziness	0.67 (0.21-2.05)	0.74 (0.2-2.72)	0.73 (0.2-2.73)
O_2_ therapy			
Nasal cannula	0.93 (0.88-0.98)	0.99 (0.93 -1.05)	1.08 (0.69-1.68)
Non-invasive ventilation	1.92 (0.58-3.52)	0.32 (0.28-2.26)	1.53 (0.64-3.64)
Mechanical ventilation	1.96 (0.21-18.34)	0.80 (0.08-7.56)	1.96 (0.31-12.29)
Laboratory markers of severity			
Platelet count <150 ×10^3^/μL	0.87 (0.46-1.63)	0.46 (0.21-0.98)	1.48 (0.84-2.63)
Lymphocyte count <1.5 ×10^3^/μL	0.78 (0.46-1.29)	0.91 (0.55-1.51)	0.89 (0.57-1.4)
D-Dimer >1 μg/mL	0.66 (0.4-1.09)	0.80 (0.47-1.37)	0.87 (0.54-1.38)
CRP >41.8 mg/L	0.64 (0.4-1.04)	0.81 (0.5-1.32)	0.85 (0.55-1.3)
Ferritin >800 ng/mL	0.85 (0.49-1.47)	0.75 (0.41-1.35)	1.14 (0.69-1.89)
Renal and electrolytes			
AKI on admission	0.47 (0.19-1.16)	0.55 (0.18-1.10)	0.97 (0.21-1.12)
K^+^ >5.2 mmol/L	1.09 (0.44-2.67)	1.15 (0.39-3.38)	0.46 (0.16-1.36)
K^+^ <3.5 mmol/L	0.52 (0.19-1.41)	0.77 (0.3-1.97)	1.28 (0.58-2.81)
ICU admission within 24 hr of hospitalization	1.65 (0.71-3.84)	2.44 (0.87-6.81)	0.86 (0.38-1.9)

ACE-I, angiotensin-converting enzyme inhibitor; ARB, angiotensin II receptor blockers; AKI, acute kidney injury; ICU, intensive care unit; CRP, C-reactive protein.

Data are presented as the odds ratio with 95% CI in parentheses.

* Assessed according to the WHO criteria.

ACE-I/ARB use was not associated with COVID-19 related hyperinflammation (based on CRP, ferritin, or D-Dimer levels), or the incidences of developing thrombocytopenia or lymphopenia (Table 2). However, ACE-I use was associated with a lower risk of developing thrombocytopenia (OR 0.46, 95% CI 0.21-0.98). Renal outcomes, including AKI and serum potassium level abnormality, were not associated with ACE-I/ARB therapy, assessed jointly or as ACE-I or ARB separately (e-Appendix, page 2, demonstrates comparisons of the mean and medians of the laboratories).

In 181 of 245 ACE-I/ARB users, therapy was continued during the non-ICU course of hospitalization, and was associated with a lower odds of ICU admission (OR 0.45, 95% CI 0.24-0.84; *P* = .012), and death (OR 0.22, 95% CI 0.09-0.56; *P* = .002), but not with mechanical ventilation (OR 0.9, 95% CI 0.33-2.64, *P* = .84). After adjusting for covariates in the multivariate regression model, the in-hospital continuation of ACE-I/ARB therapy was associated with reduced mortality (OR 0.22, 95% CI 0.073-0.67, *P* = .008).

The study patients received at least one antihypertensive agent for the treatment of hypertension (93.8%) or cardiovascular diseases (29.6%). Ninety (26.6%) patients used an ACE-I with 3.3% receiving maximum doses, whereas 155 (45.8%) patients used an ARB with 20.5% receiving maximum doses. Lisinopril and losartan were the most frequently used ACE-I (46 of 90) and ARB (71 of 155), respectively. ACE-I and ARB types and doses are presented in the e-Appendix (page 4). Developing severe COVID-19 on the day of hospitalization was not correlated with the doses of ACE-I (*r* = 0.117, *P* = .27) or ARB (*r* = 0.096, *P* = .23).

### ICU Course and Outcomes

On follow-up, 102 (30.2%) patients required ICU admission, 69 (67.6%) patients were using ACE-I/ARB on hospital admission (24 patients using ACE-I, and 45 patients using ARB). Table 3 lists the clinical characteristics and outcomes of patients who required ICU admission. There was no association of ACE-I/ARB use and risk of ICU admission or other clinical characteristics assessed on transfer to the ICU, including SOFA score, MAP <65 mmHg, PaO_2_/FiO_2_ ratio, or the need for a vasopressor, Table 4. Markers of a complicated ICU stay, including need for renal replacement therapy, developing ARDS, mechanical ventilation, or death, were not associated with the use of RAS blockers. No patient in the cohort was managed with ECMO during the study period.

**Table 3.** Clinical Characteristics and Outcomes of Patients Admitted to ICU Presented According to ACE-I and ARB Use or Not.

	Study cohort (n = 102)	ACE-I-ARB (n = 69)	Non-ACE-I/ARB (n = 33)	*P* value
ICU admission	102 (30.2%)	69 (28.2%)	33 (35.5%)	.19
ICU admission within 24 hours of hospitalization	61 (18.0%)	44 (63.8%)	17 (51.5%)	.23
SOFA score	4 (2-7)	4 (2.5-7)	4 (2-8)	.52
PaO_2_/FiO_2_ ratio	125 (90-198)	119.3 (84.2-189)	144.4 (103.7-212.2)	.18
Vasopressor on ICU admission	16 (15.7%)	10 (14.5%)	6 (18.2%)	.63
Vasopressor use during ICU stay	39 (38.6%)	29 (42.6%)	10 (30.3%)	.23
Mechanical ventilation	44 (43.1%)	30 (43.5%)	14 (42.4%)	.92
Time to mechanical ventilation, days	11 (5-17)	11 (5-16)	10 (5.5-20)	.68
ARDS	71 (69.6%)	47 (68.1%)	24 (72.7%)	.11
Severity				.52
Mild	1 (1.4%)	1 (2.1%)	0 (0%)	
Moderate	38 (53.5%)	24 (51.1%)	14 (58.3%)	
Severe	30 (42.3%)	22 (46.8%)	8 (33.3%)	
ICU stay, days	9 (5-16)	10.5 (5-17)	7 (4-13)	.13
ICU death	22 (21.6%)	15 (21.7%)	7 (21.2%)	.58

ICU, intensive care unit; SOFA, Sequential Organ Failure Assessment; PaO_2_/FiO_2_, partial pressure of oxygen in arterial blood to the fractional concentration of oxygen in inspired air; ARDS, acute respiratory distress syndrome.

**Table 4.** Associations of ACE-I/ARB Use With Various ICU Clinical Characteristics and Outcomes.

	ACE-I/ARB, n = 245, OR (95% CI)	ACE-I, n = 90, OR (95% CI)	ARB, n = 155, OR (95% CI)
Outcomes on day of admission to the ICU			
Admission to ICU	0.71 (0.42-1.18)	0.79 (0.46-1.35)	0.88 (0.55-1.41)
SOFA score	0.94 (0.84-1.06)	1.01 (0.89-1.15)	0.94 (0.84-1.06)
Vasopressors use	0.76 (0.25-2.31)	0.41 (0.08-1.97)	1.32 (0.45-3.85)
MAP <65 mmHg	1.01 (0.97-1.04)	1.00 (0.97-1.04)	1.00 (0.97-1.03)
Respiratory rate >30 breath/min	1.01 (0.95-1.06)	0.97 (0.91-1.03)	1.01 (0.95-1.06)
PaO_2_/FiO_2_ ratio	0.99 (0.99-1.00)	0.99 (0.99-1.00)	0.99 (0.99-1.00)
PaO_2_/FiO_2_ ratio of <300 mmHg	3.30 (0.52-20.7)	1.1 (0.12-11.1)	3.38 (0.36-31.4)
Outcomes during ICU stay			
Mechanical ventilation	1.04 (0.45-2.41)	0.52 (0.28-1.88)	1.29 (0.58-2.84)
ARDS	0.98 (0.38-2.50)	0.92 (0.38-2.23)	1.09 (0.38-3.13)
Vasopressor use	1.71 (0.70-4.14)	0.89 (0.36-2.41)	1.66 (0.74-3.70)
Renal replacement therapy	1.30 (0.26-6.39)	0.33 (0.04-2.72)	2.37 (0.58-9.65)
ICU stay	0.68 (0.27-1.30)	0.88 (0.21-1.03)	1.02 (0.77-1.87)
ICU death	0.63 (0.29-1.38)	0.38 (0.13-1.12)	1.22 (0.58-2.57)

ICU, intensive care unit; MAP, mean arterial pressure; PaO_2_/FiO_2_, partial pressure of oxygen in arterial blood to the fractional concentration of oxygen in inspired air ratio; ARDS, acute respiratory distress syndrome; SOFA, Sequential Organ Failure Assessment.

**Figure 1.**
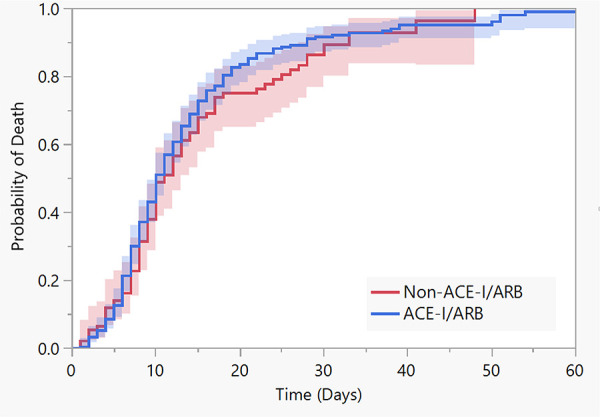
Kaplan-Meier cumulative probability of death among patients with COVID-19 receiving or not receiving ACE-I/ARB therapy. Adjusted multivariable Cox regression model for age, obesity and chronic illness testing the association of ACE-I/ARB use with the risk of death (HR 0.69, 95% CI 0.30-1.58; *P* = .35).

**Figure 2.**
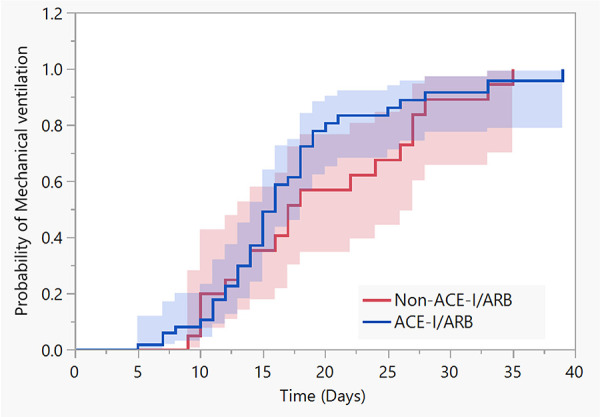
Kaplan-Meier cumulative probability of) mechanical ventilation among patients with COVID-19 receiving or not receiving ACE-I/ARB therapy. Adjusted multivariable Cox regression model for age, obesity, and chronic illness testing the association of ACE-I/ARB use and mechanical ventilation (HR 0.90, 95% CI 0.45-1.80, *P* = .77).

In total 31 (9.17%) patients from the cohort died in-hospital, of whom 9 (2.6%) died in non-ICU settings. The adjusted multivariable Cox regression model showed no association of ACE-I/ARB use and the risks of death (HR 0.69, 95% CI 0.30-1.58; *P* = .35) or mechanical ventilation (HR 0.90, 95% CI 0.45-1.80; *P* = .77) versus not using ACE-I/ARB (Figures 1 and 2). Furthermore, ACE-I/ARB therapy was not associated with the composite of mechanical ventilation and death according to an adjusted Cox regression model (HR 0.95, 95% CI 0.51-1.78; *P* = .87) versus not using ACE-I/ARB.

## Discussion

In patients with hypertension or cardiovascular disease, this multicenter prospective study showed that ACE-I/ARB therapy was not a risk factor for severe COVID-19, assessed on admission to hospital. Furthermore, ACE-I/ARB use was not associated with increase in ICU admission, mechanical ventilation, or in-hospital death. On the other hand, continuing ACE-I/ARB therapy during hospitalization for COVID-19 decreases the likelihood of mortality. To our knowledge, this is the first clinical study to prospectively assess the potential impact of ACE-I/ARB therapy on the severity of COVID-19.

A retrospective study from China assessed 1128 hypertensive patients hospitalized for COVID-19. Inpatient use of ACE-I/ARB therapy (n = 188) was associated with a lower risk of mortality compared with that of patients not using these agents.^17^ This finding is consistent with our observation of a reduced risk of mortality in patients continuing ACE-I/ARB therapy during hospitalization. The authors attributed this favorable finding to ARB amelioration of lung injury caused by the accumulation of angiotensin II secondary to SARS-CoV-2-induced ACE2 downregulation.^18^ Although this is a plausible explanation, the decreased mortality was limited to hospitalized patients in both the retrospective study and our study. The sudden cessation of chronic ACE-I/ARB therapy during hospitalization might predispose patients with COVID-19 to detrimental effects caused by an unknown mechanism.^19^

A large Danish retrospective study assessed 895 patients with COVID-19 receiving ACE-I/ARB based on data extracted from prescription filling records.^10^ In that study, patients who received ACE-I/ARB were older than those using other antihypertensive agents. After adjusting for age and sex, ACE-I/ARB use was shown to be associated with increased risk of severe COVID-19 (HR 1.32, 95% CI 1.10-1.58, *P* = .003), but not with mortality (HR 0.97, 95% CI 0.79-1.18, *P* = .82). Another population-based analysis from the UK reported no association between ACE-I/ARB use and the severity of COVID-19, assessed by ICU admission.^11^ These large-scale population-based studies shown controversial results regarding ACE-I/ARB impact on COVID-19 severity. A possible explanation for the different findings between the 2 studies is the variability of severity definitions. In our clinical study, we used the WHO interim guidance to assess COVID19 severity, which is acquiring popularity in COVID19 paradigm and has been used in many large studies.^20^

A recent meta-analysis of 16101 COVID-19 patients with a concurrent hypertension reported a mortality rate of 12.1% in ACE-I/ARB users compared to 14.5% among non-users (risk ratio 0.7; 95% CI 0.53-0.91, *P* < .007).^21^ A similar survival benefit was observed in patients continuing ACE-I/ARB therapy during hospitalization for COVID-19 in our study. The safety of ACE-I/ARB use in COVID-19 patients from a Middle Eastern country, as our study showed, is consistent with the outcomes of other studies reported from different regions of the world.^21^ Studies have overlooked examining a potential ACE-I/ARB dose-effect on COVID19 severity.^10,11,17^ Different doses of ACE-I and ARB yielded variable degrees of ACE2 expressions in animal models.^5^ We did not detect a correlation between ACE-I or ARB doses and COVID19 severity in our study. Therefore, it is unlikely that ACE-I/ARB therapy unfavorably interacts with SARS-CoV-2 at the clinically used doses.

Upregulation of ACE2 expression and activity in response RAS blockade was reported to be consistent for ARB therapy, while it was variable with ACE-I administration.^5,8^ We have examined ACE-I and ARB therapies separately; neither showed an association with COVID-19 severity, according to the WHO criteria. Moreover, we found that RAS blockers therapy was a risk factor for serious vital signs or laboratory results indicating severe COVID-19, except for a lower odds of thrombocytopenia with ACE-I use. This latter effect of ACE-I on platelet count in COVID-19 patients could be regarded as a favorable outcome, which merits further assessment by comparing ACE-I to ARB in head-to-head in COVID-19 patents.

Notably, 14 (3.1%) patients were excluded from this study for discontinuing their ACE-I/ARB therapy for fear of increased risk of severe COVID-19. Although our study was not equipped to explore this point. To our knowledge, no study has described the magnitude of discontinuing ACE-I/ARB therapy during the COVID-19 pandemic. Larger epidemiological studies could provide better insights regarding this attitude across various societies.

Recent evidence demonstrated an increased risk of hypokalemia in hospitalized COVID-19 patients.^22^ A proposed mechanism involves enhanced ACE2 degradation after binding with SARS-CoV-2; thus, the unopposed RAS axis generates increased potassium loss in infected patients.^19^ In non-COVID-19 patients, hyperkalemia is not an uncommon adverse reaction of ACE-I/ARB therapy, occurring at rates exceeding 20%.^23^ Interestingly, hyperkalemia was not associated with ACE-I/ARB use in our study. Possibly, the degradation of ACE2 by SARS-CoV-2 binding countered the potassium retaining activity of ACE-I/ARB. The mean serum potassium level was lower with ACE-I/ARB use compared to other antihypertensive agents in our study. A higher rate of use of thiazide diuretics and a lower rate of patients with chronic kidney diseases in patients receiving ACE-I/ARB could be another explanation for this observation on serum potassium level.

Our study findings provide clinical evidence regarding ACE-I and ARB therapy during the COVID-19 pandemic, in response to calls made by several medical societies to clarify the theoretical risk of harm.^9^ Our study supports their recommendation in continuing ACE-I/ARB therapy for indications where these agents are shown to be beneficial, such as heart failure, hypertension, and ischemic heart disease.^9^ Uniquely, the prospective design of our study allowed us to identify COVID-19 patients presenting to the hospital on active ACE-I and ARB therapy. We also examined the potential effect of ACE-I/ARB dosing on developing severe COVID-19 while observing changes in serum potassium level in patients with COVID-19 receiving a RAS-blocker.

Our study has several limitations. First, we did not perform sample size calculations to enable us with confidence to apply central tendency measures of ACE-I/ARB therapy versus other antihypertensive treatments. However, we conducted our study during the period of COVID-19 peak in Saudi Arabia,^24^ with 3 centers located in Riyadh, the most populated city in the Kingdom, so the convenience sample that we studied might be sufficient for addressing the primary purpose of the study. A second limitation is that we did not include medications claimed to treat COVID-19 in our analysis. The Saudi Arabian Ministry of Health performed consecutive updates on COVID-19 treatment protocols during the study period. With the exception of corticosteroid use in critically ill patients, the efficacy of various other medications used in COVID-19 management is debated.^25^ However, the primary endpoint of our study was the point of admission to hospital where the role of medications such as hydroxychloroquine is limited. A third limitation is the non-randomized allocation of antihypertensive medications in the study. Outcomes from randomized controlled trials are needed for better assessment of ACE-I/ARB effects during the COVID-19 pandemic.

In conclusion, patients with hypertension or cardiovascular diseases receiving ACE-I or ARB are not at increased risk for severe COVID-19 on admission to hospital, whilst ICU admission, mechanical ventilation, or mortality are not associated with ACE-I or ARB use before hospitalization for COVID-19. Due to a lower risk of mortality, clinicians are advised to continue prehospitalization ACE-I/ARB therapy in COVID-19 patients during hospitalization.

## Supplemental Material

Supplemental Material, sj-pdf-1-cpt-10.1177_1074248420976279 - Association of Angiotensin-Converting Enzyme Inhibitors and Angiotensin II Blockers With Severity of COVID-19: A Multicenter, Prospective Study Click here for additional data file. Supplemental Material, sj-pdf-1-cpt-10.1177_1074248420976279 for Association of Angiotensin-Converting Enzyme Inhibitors and Angiotensin II Blockers With Severity of COVID-19: A Multicenter, Prospective Study by Hakeam A. Hakeam, Muhannad Alsemari, Zainab Al Duhailib, Leen Ghonem, Saad A. Alharbi, Eid Almutairy, Nader M. Bin Sheraim, Meshal Alsalhi, Ali Alhijji, Sara AlQahtani, Mohammed Khalid and Mazin Barry in Journal of Cardiovascular Pharmacology and Therapeutics
