# Supplementary material for: Association of Angiotensin-Converting Enzyme Inhibitors and Angiotensin II Blockers With Severity of COVID-19: A Multicenter, Prospective Study
Source: J Cardiovasc Pharmacol Ther. 2020 Nov 24;26(3):244–52. doi: 10.1177/1074248420976279 (PMC8010897; doi:10.1177/1074248420976279)
Supplement: Supplemental Material, sj-pdf-1-cpt-10.1177_1074248420976279 - Association of Angiotensin-Converting Enzyme Inhibitors and Angiotensin II Blockers With Severity of COVID-19: A Multicenter, Prospective Study [file sj-pdf-1-cpt-10.1177_1074248420976279.pdf]

Supplementary Appendix:

- **Supplementary Page 1.**
  - **Table 1:** Distribution of the patient cohort according to health-care centre**.**
  - **Table 2:** Frequency of symptoms experienced by the study cohort of patients with COVID-19 admitted to four hospitals in Saudi Arabia.
- **Supplementary Page 2.**
  - **Table 3:** Laboratory results of the cohort of patients with COVID-19 with hypertension or cardiovascular disease, with comparisons of patients using ACE-I/ARB and non-users
  - Characteristics of the angiotensin converting enzyme inhibitor (ACE-I) and angiotensin II receptor blocker (ARB) therapeutics
    - **Table 4:** Type of angiotensin converting enzyme inhibitors and doses used by the study cohort
- **Supplementary Page 3.**
  - **Table 5:** Type of angiotensin II receptor antagonist and doses used by the study cohort
  - **Table 6:** Description of the angiotensin converting enzyme inhibitors and angiotensin II receptor antagonists, with combinations used by 245 patients with COVID-19 in the study
  - **Table 7:** Description of the non-angiotensin converting enzyme inhibitors and angiotensin II receptor antagonists, with combinations used by 93 patients with COVID-19 in the study
- **Supplementary Page 4.**
  - **Table 8:** COVID-19 severity outcomes of the study cohort, ACE-I/ARB users and non-users, with comparisons
  - **Table 9:** Types of oxygen therapy in the study cohort, described according to ACE-I/ARB use
- **Supplementary Page 5.**
  - **Table 10:** Characteristics of ARDS with PaO2/FiO2 ratio
  - **Table 11:** Saudi Ministry of Health (MOH) Protocol for Management of COVID-19
  - **Table 12:** King Faisal Specialist Hospital and Research Centre, Riyadh (KFSH&RC) risk stratification for patients diagnosed with COVID-19
- **Supplementary Page 6.**
  - **Table 13:** King Faisal Specialist Hospital and Research Centre, Riyadh (KFSH&RC) management protocol for patients diagnosed with COVID-19

Supplementary Table 1. Distribution of the patient cohort according to the health-care centre

Health-care centre	Number of enrolled patients n (%)	City	Sector
King Faisal Specialist Hospital and Research Centre	168 (49.7)	Riyadh, Saudi Arabia	Governmental, referral centre from around the Kingdom
King Khalid University Centre	73 (21.5)	Riyadh, Saudi Arabia	Governmental, tertiary care university hospital
Buraidah Central Hospital, Qassim	75 (22.1)	Buraidah, Saudi Arabia	Governmental, secondary care hospital
King Abdullah bin Abdulaziz University Hospital*	22 (6.5)	Riyadh, Saudi Arabia	Governmental secondary care university hospital

* Started enrolment of patients on June 10, 2020

Supplementary Table 2. Frequency of symptoms experienced by the study cohort of patients with COVID-19 admitted to 4 hospitals in Saudi Arabia:

Symptoms	King Faisal Specialist Hospital and Research Centre, Riyadh n=168	King Khalid University Hospital, Riyadh n=73	Buraidah Central Hospital, Qassim n=75	King Abdullah bin Abdulaziz University Hospital, Riyadh n= 22
**Respiratory symptoms**				
Sore throat, rhinorrhoea or anosmia, n (%)	125 (74.4%)	64 (87.6%)	59 (78.6%)	15 (68.1%)
Cough, n (%)	116 (69.0%)	54 (73.9%)	39 (52%)	13 (59.0%)
**Non-respiratory symptoms**				
Fever, n (%)	112 (66.6%)	55 (75.3%)	46 (61.3%)	10 (45.4%)
Loss of consciousness, severe dizziness, n (%)	8 (4.7%)	6 (8.2%)	0 (0%)	0 (0%)
Diarrhoea, n (%)	57 (33.9%)	23 (31.5%)	6 (8%)	4 (18.1%)
**Asymptomatic,** n (%)	10 (5.9%)	4 (5.4%)	8 (10.6%)	4 (18.1%)

One patient may have presented with more than one symptom

Cough was found to be a consistent complaint in symptomatic patients, appearing in 65.6% of the total cohort. The predominate non-respiratory symptom was fever.
No patient presented to Buridah Central Hospital and King Abdullah bin Abdulaziz University Hospital with loss of consciousness.

Supplementary Table 3: Laboratory results of the cohort of patients with COVID-19 with hypertension or cardiovascular disease, with comparisons of patients using ACE-I/ARB and non-users

**Laboratory**	**Study cohort (n=338)**	**ACE-I/ARB (n=245)**	**Non-ACE-I/ARB (n=93)**	***P* value**
D-Dimer, μg/mL	0.82 (0.51-0.13)	0.72 (0.43- 1.13)	0.92 (0.59-1.36)	0.10
Ferritin, ng/mL	569 ± 603	552.6 ± 571	615.3 ±681	0.42
CRP, mg/L	47.6 (11.0-111.5)	40 (9.3-107.8)	63 (12-150.9)	0.16
Creatinine kinase, U/L	207 ± 332	202 ± 305	219± 392	0.17
Lactate dehydrogenase, U/L	317 ± 134	312 ±124	328 ± 157	0.54
White blood cell count, $\times 10^3/\mu L$	6.4 ± 2.9	6.1 ± 3.5	7.0 ± 3.5	0.028
Lymphocyte count, $\times 10^3/\mu L$	1.24 ±0.71	1.25 ± 0.68	1.22 ± 0.79	0.68
Platelet count, $\times 10^3/\mu L$	225 ± 82	227 ± 80	220 ± 86	0.49
Serum potassium, mmol/L	4.5 ± 0.3	4.21 ± 0.57	4.3 ±0.75	0.017
Urea, mmol/L	7.4 ± 6.0	7.2 ± 5.8	8.0 ± 6.4	0.11
Serum creatinine, mmol/L	122.9 ± 126	114.6 ±111	144 ±159	0.003
Creatinine clearance, mL/min/1.73 m^2	69.9 ± 30.9	71.4 ±29.0	65 ± 34.0	0.064

ACE-I, angiotensin converting enzyme inhibitor; ARB, angiotensin II receptor blocker

Data are expressed as mean ± standard deviation and compared using the student *t*-test, or medians and interquartile range and compared with the Mann-Whitney *U* test

Characteristics of the angiotensin converting enzyme inhibitor (ACE-I) and angiotensin II receptor blocker (ARB) therapeutics

Supplementary Table 4. Type of angiotensin converting enzyme inhibitors with doses used by the study cohort

**ACE-I n=90 (%)**	**Total daily dose** n (%)*	**Mean dose (mg)**	**Maximum dose (mg)**	**Minimum dose (mg)**	**Continued during hospitalisation*** n(%)
Lisinopril n= 46 (51.1)	2.5 mg: 9 (19.5)	10.38	40	2.5	29 (63.0)
	5 mg: 13 (28.2)				
	10 mg: 13 (28.2)				
	20 mg: 9 (19.5)				
	40 mg: 2 (4.3)				
Enalapril n=22 (24.4)	2.5 mg: 1 (4.5)	7.11	20	2.5	19 (86.3)
	5 mg: 8 (36.3)				
	10 mg: 11 (50)				
	20 mg: 2 (9.09)				
Perindopril n= 16 (17.7)	1.25 mg: 1 (6.2)	5.57	10	1.25	11 (68.7)
	5mg: 10 (62.5)				
	8 mg: 1 (6.2)				
	10 mg: 4 (25)				
Captopril n=6 (6.6)	37.5 mg: 2 (33.3)	66.66	150	37.5	6 (100)
	50 mg: 2 (33.3)				
	75 mg: 1 (16.6)				
	150 mg : 1 (16.6)				

* The percentages of doses were calculated for each ACE-I
** Patients maintained on ACE-I during non-ICU hospital course, with no more than 48 hours of the antihypertensive agent being withheld

Table 5: Type of angiotensin II receptor antagonist, with doses used by the study cohort

**ARB** **n=155 (%)**	**Total daily dose** **n** (%)*	**Mean dose (mg)**	**Maximum dose (mg)**	**Minimum Dose (mg)**	**Continued during hospitalisation*** n(%)
Losartan n=72 (46.45)	25 mg: 2 (2.7)	65.62	100	25	52 (72.2)
	50 mg: 46 (63.8)				
	75 mg: 1 (1.3)				
	100mg: 23 (31.9)				
Valsartan n=43 (27.7)	40 mg: 1 (2.3)	107.46	160	40	36 (83.7)
	50 mg: 1 (2.3)				
	51 mg: 1 (2.3)				
	80 mg: 24 (55.8)				
	160 mg:16 (37.0)				
Irbesartan n=27 (17.4)	75 mg: 2 (7.4)	194.44	300	75	14 (51.8)
	150mg: 16(59.2)				
	300mg: 9 (33.3)				
Telmisartan n=9 (5.8)	40 mg: 7 (77.7)	48.88	80	40	9 (100)
	80 mg: 2 (22.2)				
Candesartan n=4 (2.5)	8 mg: 1 (25)	12	80	8	4 (100)
	16 mg: 2 (50)				
	80 mg: 1 (25)				

* The percentages of doses were calculated for each ARB
* Patients maintained on ARB during non-ICU hospital course, with no more than 48 hours of the antihypertensive agent being withheld

Supplementary Table 6: Description of the angiotensin converting enzyme inhibitors and angiotensin II receptor blockers, with combinations used by 245 patients with COVID-19 in the study

**ACE-I combinations**	
Combined antihypertensive agent	n (%)
β-blocker	27 (30)
Calcium channel blocker	21 (23.3)
Thiazide	6 (6.6)
**ARB combinations**	
β-blocker	53 (34.1)
Calcium channel blocker	69 (44.5)
Thiazide	43 (27.7)

ACE-I, angiotensin converting enzyme inhibitor; ARB, angiotensin receptor blocker

Supplementary Table 7: Description of the non-angiotensin converting enzyme inhibitors and angiotensin II receptor antagonists used by 93 patients with COVID-19 in the study

**Antihypertensive agent**	**n (%)**
β-blocker	39 (41.9)
Calcium channel blocker	74 (79.5)
Thiazide	3 (3.2)

Supplementary Table 8: COVID-19 severity outcomes of the study cohort, ACE-I/ARB users and non-users, with comparisons

	**Study cohort (n=338)**	**ACE-I/ARB (n=245)**	**Non-ACE-I/ARB (n=93)**	***P*-value**
COVID-19 severity				0.79
Non-severe	240 (71%)	173 (70.6%)	67 (72%)	
Severe	98 (29%)	72 (29.4%)	26 (28%)	
Temperature > 38 C^0	157 (46.4%)	117 (47.8%)	40 (43%)	0.43
Respiratory rate > 30 breaths per min)	51 (15.1%)	38 (15.5%)	13 (14%)	0.72
Mean arterial blood pressure < 65 mmHg	23 (6.8%)	19 (7.8%)	4 (4.3%)	0.26
Oxygen saturation < 94%	168 (49.7%)	119 (48.6%)	49 (52.7%)	0.49
Loss of consciousness or altered mental status	14 (4.1%)			
Acute kidney injury	38 (17%)	21 (38.2%)	17 (56.7%)	0.10
Need for nasal canula	211 (62.4%)	144 (59%)	66 (71%)	0.06
D-Dimer				0.10
> 1 μg/mL	105 (31.1%)	70 (28.6%)	35 (37.6%)	
≤ 1 μg/mL	233 (68.9%)	175 (71.4%)	58 (62.4%)	
Ferritin				0.56
> 800 ng/mL	80 (23.7%)	56 (22.9%)	24 (25.8%)	
≤ 800 ng/mL	258 (76.3%)	189 (77.1%)	69 (74.2%)	
C-reactive protein				0.07
> 41 mg/L	190 (56.2%)	137 (55.9%)	42 (45.2%)	
≤ 41 mg/L	148 (43.8%)	108 (44.1%)	51 (54.8%)	
Platelet count				0.66
≥ 150 $\times 10^3$ /μL	281 (83.1%)	205 (83.7%)	76 (81.7%)	
< 150 $\times 10^3$ /μL	57 (16.9%)	40 (16.3%)	17 (18.3%)	
Lymphocyte count				0.34
≥1.5 $\times 10^3$ /μL	119 (35.2%)	90 (36.7%)	29 (31.2%)	
< 1.5 $\times 10^3$ /μL	219 (64.8%)	155 (63.3%)	64 (68.8%)	
Serum potassium level				
< 3.5 mmol/L	27 (8%)	20 (8.2%)	7 (7.5%)	0.847
> 5.2 mmol/L	17 (5%)	10 (4.1%)	7 (7.5%)	0.19
Hospitalisation, days	10 (7-16.5)	10 (7-15)	10 (7.25-16)	0.64

ACE-I, angiotensin converting enzyme inhibitor; ARB, angiotensin II receptor blocker.

Data are expressed as percentages and compared using χ^2 test or Fisher's test for categorical data, and medians and interquartile range and compared with the Mann-Whitney *U* test

Supplementary Table 9: Types of oxygen therapy in the study cohort, and described according to ACE-I/ARB use:

**Oxygen Therapy,** n (%)	**Cohort n= 338 (%)**	**ACE-I/ARB users n= 245 (%)**	**Non-ACE-I/ARB users n= 93 (%)**	***P*-value**
Nasal cannula/ facemask	207 (61.2)	142 (57.9)	65 (69.8)	0.2
Non-invasive ventilation (CPAP, BIPAP)	30 (8.8)	21 (8.57)	9 (9.6)	0.71
Mechanical ventilation	44 (43.1)	30 (43.5)	14 (42.4)	0.61

ACE-I, angiotensin converting enzyme inhibitor; ARB, angiotensin II receptor blocker; CPAP, continuous positive airway pressure; BIPAP, bilevel positive airway pressure

Supplementary Table 10: Characteristics of ARDS with PaO2/FiO2 ratio

**ARDS*<br>n= 74**	**Mean PaO_2/FiO_2 ratio**	**Lowest PaO_2/FiO_2 ratio**
Mild<br>n=1 (1.35%)	212	212
Moderate<br>n =40 (54.05%)	139.99	40
Severe<br>n= 33 (44.59%)	93.52	41

*ARDS, Acute Respiratory Distress Syndrome; PF Ratio: Horowitz Index

Supplementary Table 11: Saudi Ministry of Health (MOH) protocol for management of COVID-19*

**Severity**	**Management**
**Asymptomatic**	Supportive care
**Mild-Moderate**	• *Triple combination therapy:* Lopinavir /Ritonavir, Ribavirin and interferon beta-1b for 14-days.<br>OR<br>• Favipiravir<br>OR<br>• Hydroxychloroquine
**Severe**	• *Triple combination therapy:* Lopinavir /Ritonavir, Ribavirin and interferon beta-1b for 14-days.<br>OR<br>• Favipiravir<br>OR<br>• Hydroxychloroquine<br>AND<br>• Dexamethasone
**Critical**	• Remdesivir<br>OR<br>• Favipiravir<br>AND<br>• Dexamethasone

*Abridged version; Last updated June 2020; Accessed September 19th 2020; Full protocol available at: https://www.moh.gov.sa/en/Ministry/MediaCenter/Publications/Pages/covid19.aspx

Supplementary Table 12: King Faisal Specialist Hospital and Research Centre, Riyadh (KFSH&RC) risk stratification for patients diagnosed with COVID-19*.

**Definition**	**Criteria**
**Asymptomatic (Stage A)**	Patients with no signs or symptoms of infection
**Mild Infection (Stage B)**	Patients with upper respiratory tract infection symptoms and other mild symptoms (including fever and gastrointestinal symptoms) without evidence of pneumonia
**Moderate Infection (Stage C)**	Patients with hypoxia with oxygen saturation less than 93% at rest or presence of pneumonia not requiring ICU admission
**Severe Infection (Stage D)**	Patients with pneumonia requiring ICU admission or any of the following:<br>1. Respiratory rate of 30 breaths/min (for paediatrics, respiratory rate more than 2 standard deviations with clinical evidence of increase work of breathing)<br>2. Arterial oxygen partial pressure to fractional inspiratory oxygen ratio (PaO2/FiO2) less than 300<br>3. More than 50% lung involvement on imaging within 24-48 hours<br>4. Critical respiratory failure requiring mechanical ventilation, septic shock or multi-organ dysfunction

*Accessed September 19th 2020; Established May 2020 by King Faisal Specialist Hospital and Research Centre, Riyadh (KFSH&RC).

Supplementary Table 13: King Faisal Specialist Hospital and Research Centre, Riyadh (KFSH&RC) management protocol for patients diagnosed with COVID-19*.

Stage	Management Protocol
**Stage A**	• No need for treatment<br>• Apply infection control measures and supportive care, if needed
**Stage B**	• Consult Infectious Diseases team<br>• Azithromycin for 5 days<br>• Hydroxychloroquine for 5 days
**Stage C**	• Consult Infectious Diseases team<br>• Azithromycin for 5 days<br>• Hydroxychloroquine for 5 days<br>• Decision to add lopinavir/ritonavir or chloroquine for 6-10 days should be made by Infectious Diseases team.<br>• In patients with suspected bacterial infection, start ceftriaxone in addition to oseltamivir for confirmed influenza<br>• May consider levofloxacin instead of ceftriaxone in severe beta lactam allergy (closely monitor QTc interval)<br>• Supportive care as needed
**Stage D**	• Azithromycin for 5 days<br>• Hydroxychloroquine for 5 days<br>• Decision to add lopinavir/ritonavir or chloroquine for 6-10 days should be made by Infectious Diseases team.<br>• The addition of high dose intravenous immunoglobulin (IVIG) 25 grams per day for 5 days for clinically deteriorating patients; should be decided by Infectious Diseases team.<br>• May consider adding tocilizumab for patients meeting eligibility criteria.<br>• In patients with suspected bacterial infection, start piperacillin/tazobacatm +/- vancomycin if MRSA† risk factors identified in addition to oseltamivir for confirmed influenza.<br>• May consider levofloxacin instead of piperacillin/tazobactam in severe beta lactam allergy (closely monitor QTc interval)<br>• There is accumulating evidence that suggests that COVID-19 may predispose to both venous and arterial thromboembolisms in critically ill patients due to excessive inflammation, hypoxia, immobilisation, and diffuse intravascular coagulation. Use of anticoagulation should be assessed on a case-by-case basis.<br>• Supportive care as needed

†Methicillin-resistant *Staphylococcus aureus*

*Accessed September 19th 2020; Established May 2020 by King Faisal Specialist Hospital and Research Centre, Riyadh (KFSH&RC).
